# An Autophagy Inducing Triterpene Saponin Derived from *Aster koraiensis*

**DOI:** 10.3390/molecules24244489

**Published:** 2019-12-07

**Authors:** Jaeyoung Kwon, Keebeom Ko, Lijun Zhang, Dong Zhao, Hyun Ok Yang, Hak Cheol Kwon

**Affiliations:** 1 Natural Product Informatics Research Center, Korea Institute of Science and Technology (KIST) Gangneung Institute, Gangneung 25451, Korea; kjy1207@kist.re.kr (J.K.); gogiup0218@kist.re.kr (K.K.); 2 Natural Product Research Center, Korea Institute of Science and Technology (KIST) Gangneung Institute, Gangneung 25451, Korea; 512515@kist.re.kr (L.Z.); 614003@kist.re.kr (D.Z.); 3 Division of Bio-medical Science and Technology, KIST School, Korea Institute of Science and Technology (KIST), Seoul 02792, Korea

**Keywords:** *Aster koraiensis*, triterpene saponin, autophagy, natural products

## Abstract

Autophagy is an important self-degradative mechanism that plays a key role in treating neurodegeneration diseases. This research aimed at discovering bioactive compounds from *Aster koraiensis*. A new triterpene saponin, astersaponin I (**1**), was isolated from the EtOH extract of *A. koraiensis*. The structure of **1** was characterized by spectroscopic methods, ECD calculation, and acid hydrolysis. The biochemical analysis showed that compound **1** significantly increased the expression of microtubule-associated protein 1A/1B light chain 3B (LC3-II) expression in SH-SY5Y cells, which indicates the induction of autophagy. Thus, further study may be needed to clarify whether compound **1** exerts beneficial effects on neurodegeneration diseases like Parkinson’s disease through autophagy induction.

## 1. Introduction

*Aster koraiensis* Nakai (Korean starwort), belonging to the family Asteraceae, is a perennial herb originating in Korea and mainly distributed in temperate regions of the Korean peninsula and Jeju Island. This plant has long been utilized for decoration, food ingredients, and traditional medicines. Its young leaves and stems are edible, and the roots have been used for the treatment of chronic bronchitis, pertussis, and pneumonia [1]. Previous phytochemical studies demonstrated that it contains polyacetylene [2,3], benzofuran [4], and sesquiterpenoids [5], which are associated with several biological activities, such as cytotoxicity and immune diseases [2,3].

Autophagy, a self-degradative process, is considered to be a crucial survival mechanism. Autophagy not only regulates the balance of energy sources and offers an adaption for nutrient stress, it also promotes the degradation of damaged cytosolic components or organelles before they become toxic to cells [6,7,8]. Autophagy is very important for the treatment of various diseases, including neurodegeneration diseases [9]. A decline of autophagy results in the deposition of toxic components in cytoplasm and finally leads to neurodegenerative diseases like Parkinson’s disease (PD) [10,11]. During the autophagy process, microtubule-associated protein light chain 3 (LC3) is conjugated to phosphatidylethanolamine to form LC3-II, which can be found on the membranes of autophagosomes. Since the formation of LC3-II reflects the amount of autophagosomes, LC3 has been regarded as the most important marker to monitor autophagy induction [12,13].

In a search to discover an autophagy-inducing molecule, we confirmed by using western blot analysis that the EtOH extract of *A. koraiensis* induced autophagy in SH-SY5Y cells; the extract (12.5, 25, and 50 μg/mL) significantly increased LC3-II expression in a dose-dependent manner. Subsequently, a bioassay-guided fractionation indicated that the *n*-BuOH fraction (12.5, 25, and 50 μg/mL) up-regulated LC3-II in a dose-dependent manner, while the fractions of n-hexane and EtOAc showed a relatively weak autophagy inducing effect. This led to the isolation and structural characterization of a biologically active compound.

## 2. Results and Discussion

### 2.1. Structural Elucidation of Astersaponin I

**Figure 1:**
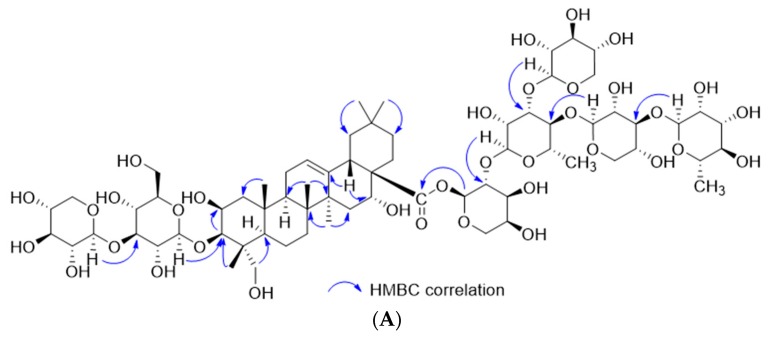

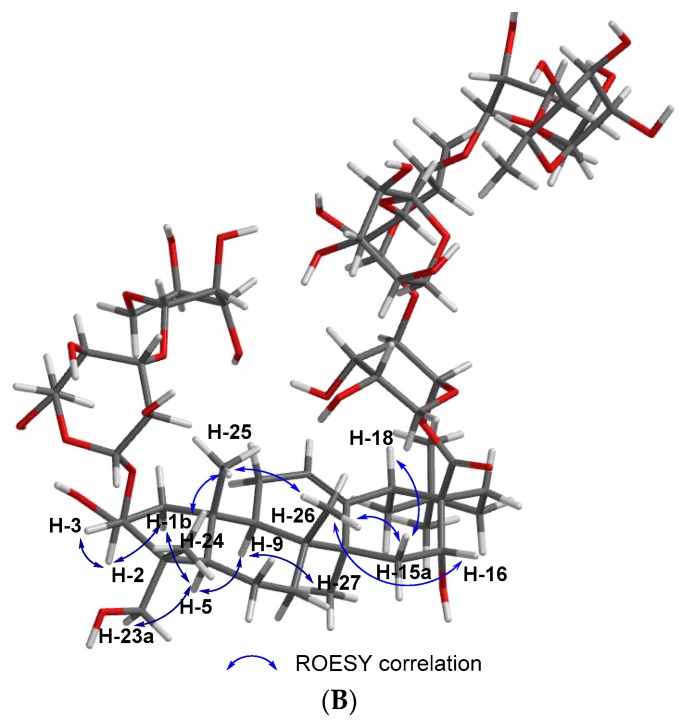
Key HMBC (**A**) and ROESY (**B**) correlations of astersaponin I (**1**).

Astersaponin I (**1**) was isolated as a white amorphous powder. The IR data exhibited absorbance bands at 3368 and 1657 cm^−1^, which indicate hydroxy and carbonyl groups, respectively (Figure S1, Supplementary Materials). The UV data displayed only terminal absorption at 205 nm (Figure S2), which was attributable to terpene with little conjugation. The molecular formula was deduced to be C_68_H_110_O_35_ on the basis of HR-MS data, and the fragmentation patterns (*m*/*z* 1487, 1355, 1209, 1077, 945, 799, 667, and 505) suggested that the triterpene aglycone was present with seven sugar moieties (Figure S3). The ^1^H- and ^13^C-NMR data exhibited characteristic signals for aglycone and sugar moieties (Table 1). Six distinct methyl singlets (*δ*_H_ 1.38, 1.31, 1.27, 0.98, 0.95, 0.89, and 0.80) and an olefinic methine signal (*δ*_H_ 5.38) were observed in the ^1^H-NMR spectrum (Figure S4), along with three resonances (*δ*_C_ 180.1, 144.7, and 123.7) in the ^13^C-NMR spectrum, which were indicative of an oleanane-type triterpenoid (Figure S5) [14]. Furthermore, two oxymethine signals (*δ*_H_ 4.49 and 4.33) and one oxymethylene signal (*δ*_H_ 3.63 and 3.24) were observed, and HSQC, COSY, and HMBC correlations indicated that two oxymethine groups were placed at C-2 and C-16, while an oxymethylene group was placed at C-23 (Figure 1A and Figures S6–S8). Consequently, the aglycone was determined to be a polygalacic acid [15]. The relative configuration of the aglycone was deduced by ROESY correlations and this NMR data was compared with that of previous reports (Figure 1B and Figure S9).

**Figure 2:**
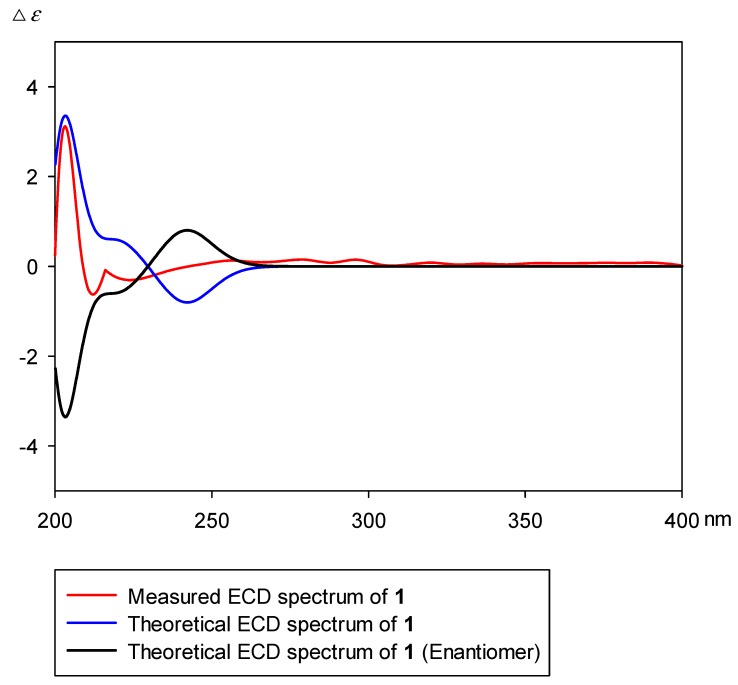
Measured and theoretical ECD spectra of astersaponin I (**1**).

**Figure 3:**
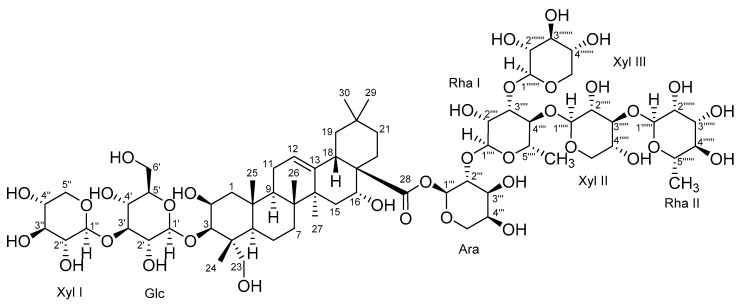
Chemical structure of astersaponin I (**1**).

The absolute configuration was determined by using ECD calculation. The measured CD spectrum of compound 1 exhibited a positive cotton effect (CE) at 203 nm (Δε = +3.1). This cotton effect is similar to that (Δε = +1.84 at 209 nm) of 2β,3β,16β,23-tetrahydroxy-olean-12-en-28-oic acid methyl-ester (methyl-polygalacate) [16,17]. The measured circular dichroism spectrum of compound 1 was well fitted with that of the theoretical ECD spectrum (Figure 2). In addition, seven characteristic peaks for anomeric protons were observed in a range between 4.40 and 5.70 ppm (*δ*_H_ 5.63 (br d, *J* = 3.0 Hz), 5.14 (br d, *J* = 1.5 Hz), 5.00 (br d, *J* = 1.5 Hz), 4.74 (br d, *J* = 8.0 Hz), 4.51 (br d, *J* = 7.5 Hz), 4.50 (br d, *J* = 7.5 Hz), and 4.49 (br d, *J* = 7.5 Hz)), which were correlated with seven anomeric carbons (*δ*_C_ 93.8, 102.8, 101.3, 105.1, 106.2, 106.3, and 105.3). These coupling constants and chemical shifts suggest that seven sugar moieties were *α*-arabinose (Ara), two were *α*-rhamnose (Rha I and Rha II), and three were *β*-xylose (Xyl I, Xyl II, and Xyl III) and *β*-glucose (Glc) moieties. The TOCSY and HSQC–TOCSY correlations enabled the grouping and overall assignment of the ^1^H and ^13^C NMR signals of each sugar moiety (Figures S10 and S11). The approximate sequence of linkages was deduced by HR-MS/MS data (Figure S3). The downfield shifts in the ^1^H-NMR spectrum and HMBC correlations from anomeric protons to relevant carbons confirmed the exact position and sequence of sugar moieties (Figure 2A). According to a previous report, the structure of **1** was similar to that of conyzasaponin K [18], except for the replacement of the β-apiose moiety by β-xylose. Acid hydrolysis and comparative studies with standard samples using HPLC demonstrated that these sugar units were l-arabinose, l-rhamnose, d-xylose, and d-glucose (Figure S12). Consequently, the structure was determined to be 3-*O*-*β*-d-xylopyranosyl-(1→3)-*β*-d-glucopyranosylpolygalacic acid-28-*O*-*α*-l-rhamnopyranosyl-(1→3)-*β*-d-xylopyranosyl-(1→4)-[*β*-d-xylopyranosyl-(1→3)]-*α*-l-rhamnopyranosyl-(1→2)-*α*-l-arabinopyranosyl-ester, which is called astersaponin I (Figure 3).

### 2.2. Bioactivities of Atersaponin I

**Figure 4:**
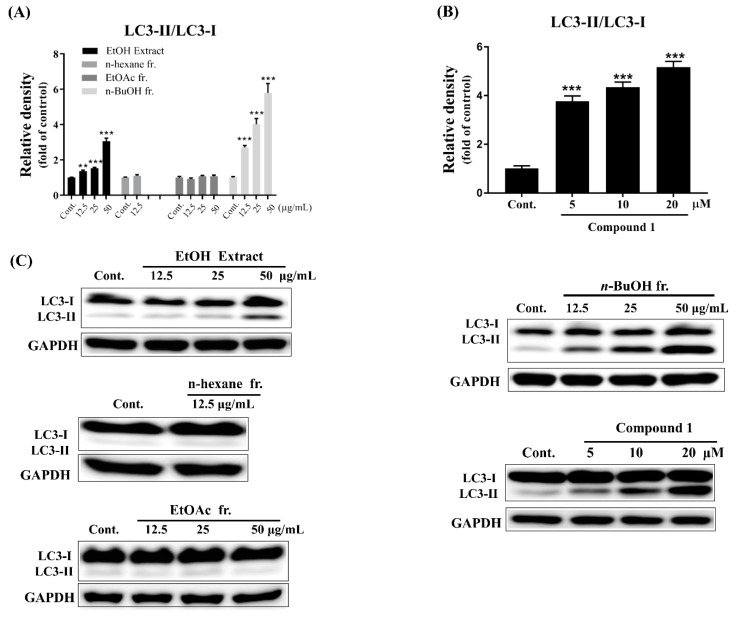
(**A**) The LC3-II expression patterns upon treatment of EtOH extract (12.5, 25, and 50 μg/mL), *n*-hexane (12.5 μg/mL), EtOAc (12.5, 25, and 50 μg/mL), and *n*-BuOH (12.5, 25, and 50 μg/mL) fractions. (**B**) The up-regulated LC3-II expression in dose-dependent manner by treatment with compound 1. (**C**) The representative western blot bands of protein marker LC3 in SH-SY5Y cells treated with EtOH extract, fractions (*n*-hexane, EtOAc *n*-BuOH), and compound 1. Data are expressed as mean ± SEM (*n* = 3). ** *p* < 0.01, *** *p* < 0.001 significant difference from control.

The extract, fractions, and astersaponin I (**1**) was assessed for its enhancing effect on autophagy by analyzing the LC3-II/LC3-I ratio in SH-SY5Y cells. The LC3-II/LC3-I ratio has extensively been used as an indicator of autophagy activation because conversion from LC3-I to LC3-II is a necessary process for autophagosome formation [19]. As shown in Figure 4, treatment with EtOH extract and n-BuOH fraction significantly increased ratio of LC3- II/LC3- I with a dose-dependent manner, while n-hexane and EtOAc fractions did not show an effect on LC3 expression (Figure 4A). Interestingly, treatment of **1** (Figure 4B) led to an increase in the LC3-II/LC3-I ratio in a dose-dependent manner, signifying the extent of autophagosome formation and autophagy activation. Previous reports have shown that several triterpene saponins, including ginsenosides, could enhance autophagy in a few cell lines, which are mainly related to cancer [20,21]. However, autophagy can also play an important role in modulating various neurodegenerative diseases like PD [10,11]. Therefore, further mechanistic studies will be needed to clarify whether compound **1** exerts a protective effect on PD through autophagy induction.

Astersaponins are known to exert antitumor, expectorant, and antitussive activities [22], while no autophagy inducing effects of astersaponins or conyzasaponins in tumor cells or neuronal cells have been reported. Furthermore, there is no previous literature on astersaponins isolated from *Aster koraiensis*. Astersaponins have been mainly reported from *Aster tataricus* [22]. Astersaponin I is the first reported saponin from *A. koraiensis.* The autophagy-inducing constituent of this plant was also reported for the first time in this paper. Even our data showed compound **1** significantly enhanced LC3-II expression, it was not enough to demonstrate autophagy was induced. Further study is needed to monitor the autophagic flux in the presence and absence of autophagy inhibitor.

## 3. Materials and Methods

### 3.1. General Experimental Procedures

Optical rotations were acquired with a Perkin-Elmer (Waltham, MA, USA) 343 polarimeter. UV and IR spectra were acquired with a Perkin-Elmer Lambda 35 spectrophotometer and a Thermo (Waltham, MA, USA) iS50 spectrometer, respectively. ECD spectra were obtained with an Applied Photophysics (Leatherhead, England) Chirascan V100 spectrometer. NMR spectra were recorded on a Varian (Palo Alto, CA, USA) 500 MHz, a Jeol (Tokyo, Japan) 600 MHz, and a Bruker (Billerica, MA, USA) 850 MHz NMR spectrometer. NMR spectrometer. HRMS data were collected on a Thermo Q-Exactive mass spectrometer. Preparative HPLC system utilized YMC (Kyoto, Japan) LC-Forte/R and an ELS detector with a Phenomenex (Torrance, CA, USA) Luna C_18_ column (10 μm, 250 mm × 21.2 mm). Column chromatography was carried out using GE Healthcare (Chicago, IL, USA) Sephadex LH-20 gel.

### 3.2. Plant Materials

The whole plant of *Aster koraiensis* was collected in October 2016 after cultivation at Pyeongchang Wild Plant Nursery and Farming Corporation (Pyeongchang, Republic of Korea). A voucher specimen (No. BS0083A1) was deposited in the Korea Institute of Science and Technology Gangneung Institute. 

### 3.3. Extraction and Isolation

The dried *A. koraiensis* (15 kg) was ground and extracted with 95% EtOH at 65 °C for 3 h. The extracted solution was evaporated in vacuo to obtain a powdered extract (1.7 kg, yield 11.3%), which was subsequently partitioned using *n*-hexane, EtOAc, and *n*-BuOH, resulting in three kinds of fractions (149 g, 175 g, and 190 g). According to biological evaluation, the *n*-BuOH fraction (1.4 g) was chromatographed using preparative HPLC under isocratic conditions (CH_3_CN/H_2_O, 7:18, flow rate 10.0 mL/min) to obtain a bioactive fraction (*t*_R_ = 33.0 min). The obtained fraction was separated on a Sephadex LH-20 column (2.8 cm × 100 cm, CH_3_OH, flow rate 0.25 mL/min) to astersaponin I (**1**, 34.6 mg, *t*_R_ = 800 min).

*Astersaponin I* (**1**): white powder; [α]^20^_D_ = −18.0 (*c* = 0.01, CH_3_OH); IR *ν*_max_ (ATR) 3392, 2916, 2850, 1637, 1571, 1416, 1088 cm^−1^; ECD (*c* = 0.1 mM, CH_3_CN) Δ*ε* = +3.1 (203); for ^1^H- and ^13^C-NMR, see Table 1; HRESIMS *m*/*z* 1487.68823 [M + H]^+^ (calcd. for C_68_H_111_O_35_, 1487.69004).

### 3.4. ECD Calculation

All computational methods, including conformational distribution, optimization, and energy calculation, were performed according to previous reports [22], which are detailed in the Supporting Information.

### 3.5. Autophagy Induction Assay

To investigate the autophagy inducing effect of *A. koraiensis*, human neuroblastoma (SH-SY5Y) cells were cultivated in six well plates at a density of 8 × 10^5^ cells/well in 2 mL DMEM medium (Gibco) at 37 °C in a humidified atmosphere with 5% CO_2_. After incubation for 24 h, the cells were treated with an EtOH extract (12.5, 25, and 50 μg/mL), *n*-BuOH fraction (12.5, 25, and 50 μg/mL), and compound **1** (5, 10, and 20 μM), respectively. After 24 h, the cells were harvested and lysed using a RIPA lysis buffer (cell signaling). The protein expression of LC3-II in cell lysates was measured using western blot analysis. A rabbit anti-LC3B primary antibody and a goat anti rabbit horseradish peroxidase-conjugated IgG secondary antibody from cell signaling were used to detect LC3 expression. The immune-blots were visualized by an ECL detection kit and analyzed by a LAS-4000 mini system (Fujifilm).

## Figures and Tables

**Table 1:** ^1^H-NMR and ^13^C-NMR spectroscopic data (500 MHz) of astersaponin I (**1**) measured in CD_3_OD ^a^.

Position	δ_C_	δ_H_ (*J* in Hz)	Intensities		Position	δ_C_	δ_H_ (*J* in Hz)
		Aglycone					Sugar Moiety
1	44.6	2.09 (dd) *J* = 14.0 Hz, 2.0 Hz	2H	Glc	1′	105.3	4.49 (d) *J* = 7.5 Hz
		1.18 (dd) *J* = 14.0 Hz, 3.5 Hz			2′	74.7	3.48 (m)
2	71.3	4.33 (m)	1H		3′	88.1	3.52 (m)
3	84.2	3.63 (m)	1H		4′	71.1	3.51 (m)
4	43.3				5′	77.5	3.31 (m)
5	48.5	1.33 (m)	1H		6′	62.3	3.81 (m)
6	18.9	1.50 (m)	2H				3.71 (m)
7	34.0	1.67 (m)	2H	Xyl I	1′′	106.2	4.51(d) *J* = 7.5 Hz
		1.35 (m)			2′′	73.2	3.64 (m)
8	41.0		1H		3′′	76.3	3.23 (m)
9	48.7,	1.63 (m)	1H		4′′	70.2	3.81 (m)
10	37.7				5′′	67.6	3.87 (d) *J* = 11.5 Hz
11	24.8	2.00, (m)	2H				3.57 (d) *J* = 11.5 Hz
		1.96 (m)		Ara	1′′	94.1	5.63 (br d) *J* = 3.0 Hz
12	123.9	5.38, (br t) *J* = 3.5 Hz	1H		2′′′	75.6	3.78 (dd) *J* = 5.0 Hz, 3.0 Hz
13	144.9				3′′′	70.6	3.91 (m)
14	43.1				4′′′	66.8	3.84 (m)
15	36.5	1.78 (m)	2H		5′′′	63.4	3.92 (m)
		1.39 (m)					3.49 (m)
16	74.8	4.49 (d) *J* = 5.0 Hz	1H	Rha I	1′′′′	101.0	5.00 (br d) *J* = 1.5 Hz
17	50.5				2′′′′	72.3	4.07 (m)
18	42.3	3.06 (br dd) *J*=14.0 Hz, 4.0 Hz	1H		3′′′′	82.7	3.87 (m)
19	47.8	2.28 (br dd) *J* = 14.0 Hz, 12.5 Hz	2H		4′′′′	78.9	3.69 (m)
		1.04 (br dd) *J* = 12.5Hz, 3.5 Hz			5′′′′	69.2	3.71 (m)
20	31.5				6′′′′	18.5	1.27 (d) *J* = 6.0 Hz
21	36.6,	1.93 (m)	2H	Xyl II	1′′′′′	105.0	4.74 (d) *J* = 8.0 Hz
		1.16 (m)			2′′′′′	75.4	3.29 (m)
22	32.1	1.92 (m)	2H		3′′′′′	84.5	3.41 (m)
		1.80 (m)			4′′′′′	70.4	3.50 (m)
23	66.0	3.63 (m)	2H		5′′′′′	67.1	3.86 (m)
		3.24 (m)					3.20 (m)
24	15.0	0.95 (s)	3H	Rha II	1′′′′′′	102.8	5.14 (br d) *J* = 1.5 Hz
25	17.8	1.31 (s)	3H		2′′′′′′	72.4	3.93 (m)
26	18.2	0.80 (s)	3H		3′′′′′′	72.4	3.70 (m)
27	27.5	1.38 (s)	3H		4′′′′′′	74.1	3.40 (m)
28	177.2				5′′′′′′	70.1	4.02 (m)
29	25.3	0.98 (s)	3H		6′′′′′′	18.0	1.24 (d) *J* = 6.0 Hz
30	33.5	0.89 (s)	3H	Xyl III	1′′′′′′′	106.3	4.50 (d) *J* = 7.5 Hz
					2′′′′′′′	75.4	3.28 (m)
					3′′′′′′′	77.8	3.32 (m)
					4′′′′′′′	71.1	3.50 (m)
					5′′′′′′′	67.3	3.91 (m)
							3.25 (m)

^a^ The respective chemical shifts in ppm are indicated by δ. Multiplicities are indicated by s (singlet), d (doublet), and m (multiplet) including coupling constant *J* in Hz.

## Supplementary Materials

The following are available online at https://www.mdpi.com/1420-3049/24/24/4489/s1. Figure S1: The IR spectrum of astersaponin I (**1**), Figure S2: The UV spectrum of astersaponin I (**1**) (CH_3_OH), Figure S3: HR-MS spectrum of astersaponin I (**1**), Figure S4: The ^1^H-NMR spectrum of astersaponin I (**1**) (500 MHz, CD_3_OD), Figure S5: The ^13^C-NMR spectrum of astersaponin I (**1**) (125 MHz, CD_3_OD), Figure S6: The ^1^H-^1^H COSY NMR spectrum of astersaponin I (**1**) (500 MHz, CD_3_OD), Figure S7: The HSQC NMR spectrum of astersaponin I (**1**) (500 MHz, CD_3_OD), Figure S8: The HMBC NMR spectrum of astersaponin I (**1**) (500 MHz, CD_3_OD), Figure S9: The ROESY NMR spectrum of astersaponin I (**1**) (500 MHz, CD_3_OD), Figure S10: The ^1^H-^1^H TOCSY NMR spectrum of astersaponin I (**1**) (600 MHz, CD_3_OD), Figure S11: The TOCSY-HSQC NMR spectrum of astersaponin I (**1**) (850 MHz, CD_3_OD), Figure S12: Sugar determination of astersaponin I (**1**), S13: ECD calculation method.
