# Supplementary material for: An Autophagy Inducing Triterpene Saponin Derived from Aster koraiensis
Source: Molecules. 2019 Dec 7;24(24):4489. doi: 10.3390/molecules24244489 (PMC6943805; doi:10.3390/molecules24244489)
Supplement: Supplementary file 1 [file molecules-24-04489-s001.pdf]

Supplementary Data

An Autophagy Inducing Triterpene Saponin Derived from *Aster koraiensis*

Jaeyoung Kwon [1,†], Keebeom Ko [1,†], Lijun Zhang [2], Dong Zhao [2], Hyun Ok Yang [2,3,*] and Hak Cheol Kwon [1,*]

[1] Natural Product Informatics Research Center, Korea Institute of Science and Technology (KIST) Gangneung Institute, Gangneung 25451, Republic of Korea; kjy1207@kist.re.kr (J.K.); gogiup0218@kist.re.kr (K.K); hkwon@kist.re.kr (H.C.K.)

[2] Natural Product Research Center, Korea Institute of Science and Technology (KIST) Gangneung Institute, Gangneung 25451, Republic of Korea; 512515@kist.re.kr (L.Z); 614003@kist.re.kr (D.Z); hoyang@kist.re.kr (H.O.Y.)

[3] Division of Bio-medical Science and Technology, KIST School, Korea Institute of Science and Technology (KIST), Seoul 02792, Republic of Korea

* Correspondence: hkwon@kist.re.kr; Tel.: +82-33-650-3504; hoyang@kist.re.kr; Tel.: +82-33-650-3501

† These authors contributed equally to this work.

Received: 30 October 2019; Accepted: 4 December 2019; Published: date

List of Supporting Information

Figure S1. The IR spectrum of compound **1**.

Figure S2. The UV spectrum of compound **1** (CH_3OH).

Figure S3. HR-MS spectrum of compound **1**.

Figure S4. The ^{1}H NMR spectrum of compound **1** (500 MHz, CD_3OD).

Figure S5. The ^{13}C NMR spectrum of compound **1** (125 MHz, CD_3OD).

Figure S6. The ^{1}H-^{1}H COSY spectrum of compound **1** (500MHz, CD_3OD).

Figure S7. The HSQC spectrum of compound **1** (500 MHz, CD_3OD).

Figure S8. The HMBC spectrum of compound **1** (500 MHz, CD_3OD).

Figure S9. The 2D ROESY spectrum of compound **1** (500 MHz, CD_3OD).

Figure S10. The 2D TOCSY spectrum of compound **1** (600 MHz, CD_3OD.)

Figure S11. The TOCSY-HSQC spectrum of compound **1** (850 MHz, CD_3OD)

Figure S12. Sugar determination of compound **1**.

S13. ECD calculation method.

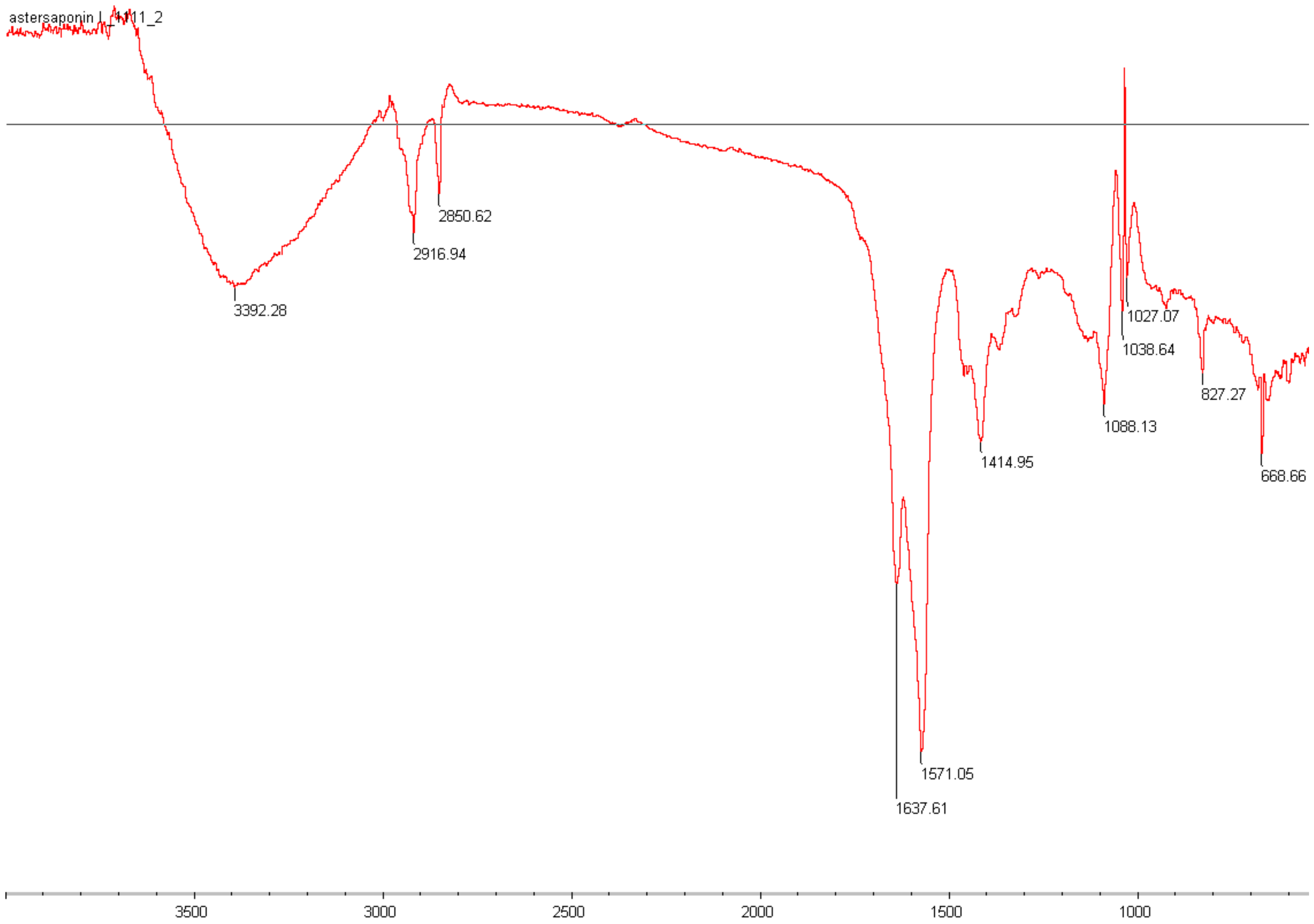

Figure S1. The IR spectrum of compound **1**.

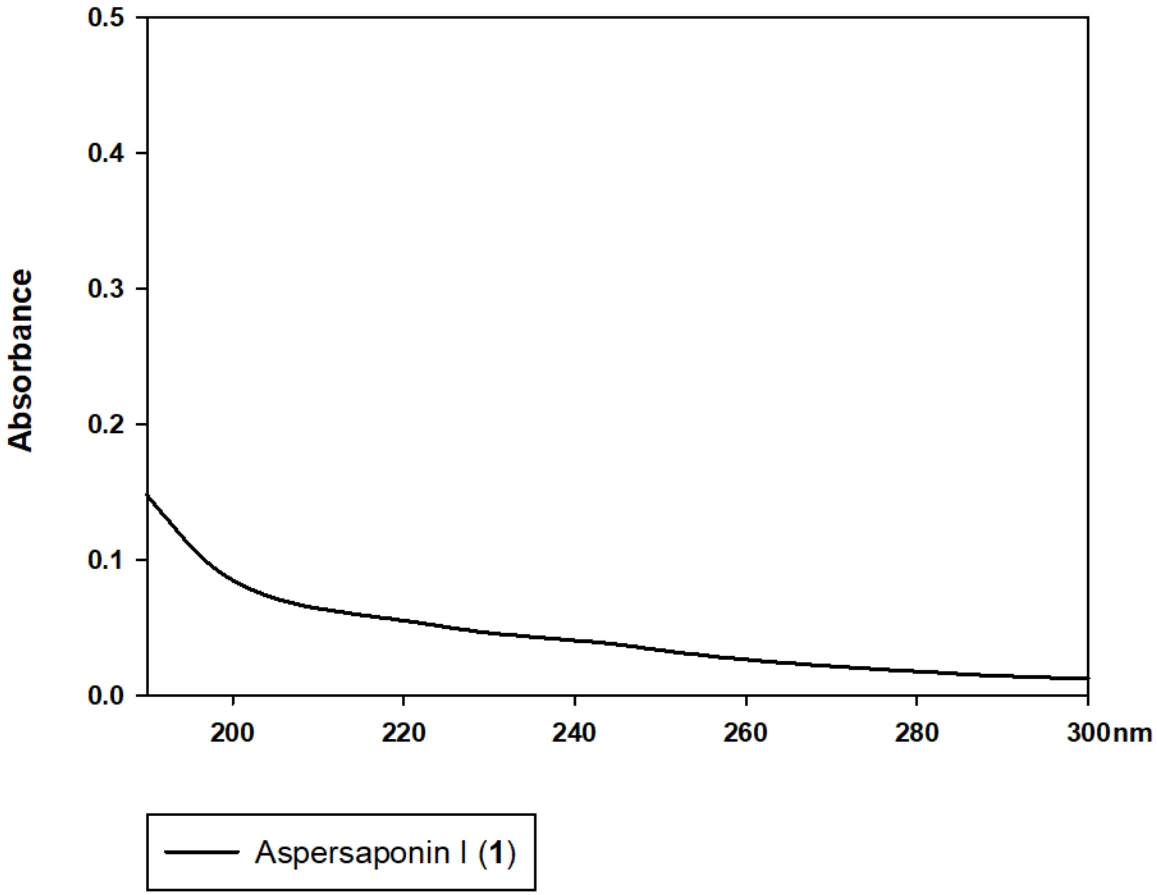

Figure S2. The UV spectrum of compound **1** (CH_3OH).

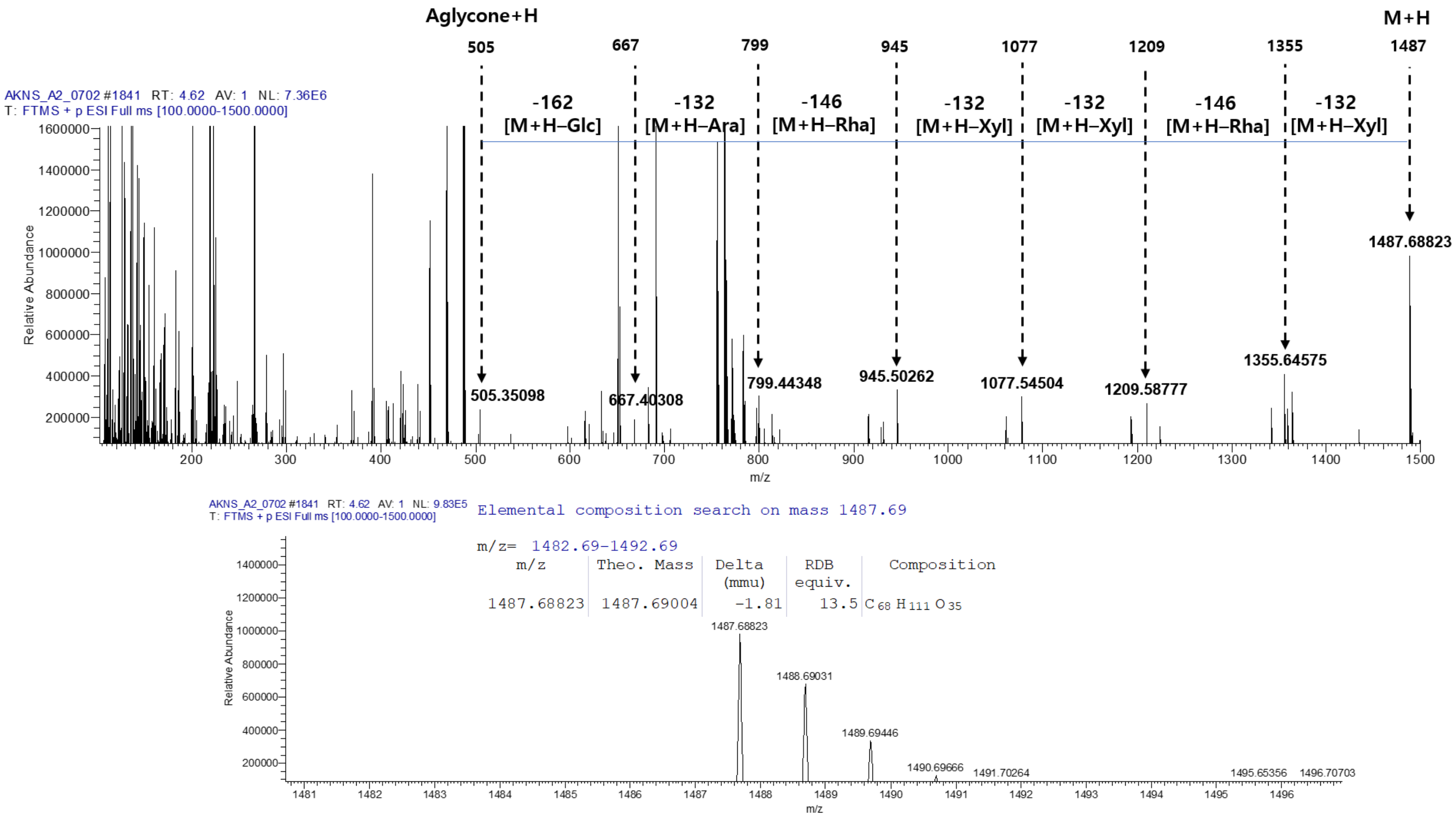

Figure S3. HR-MS spectrum of compound **1**.

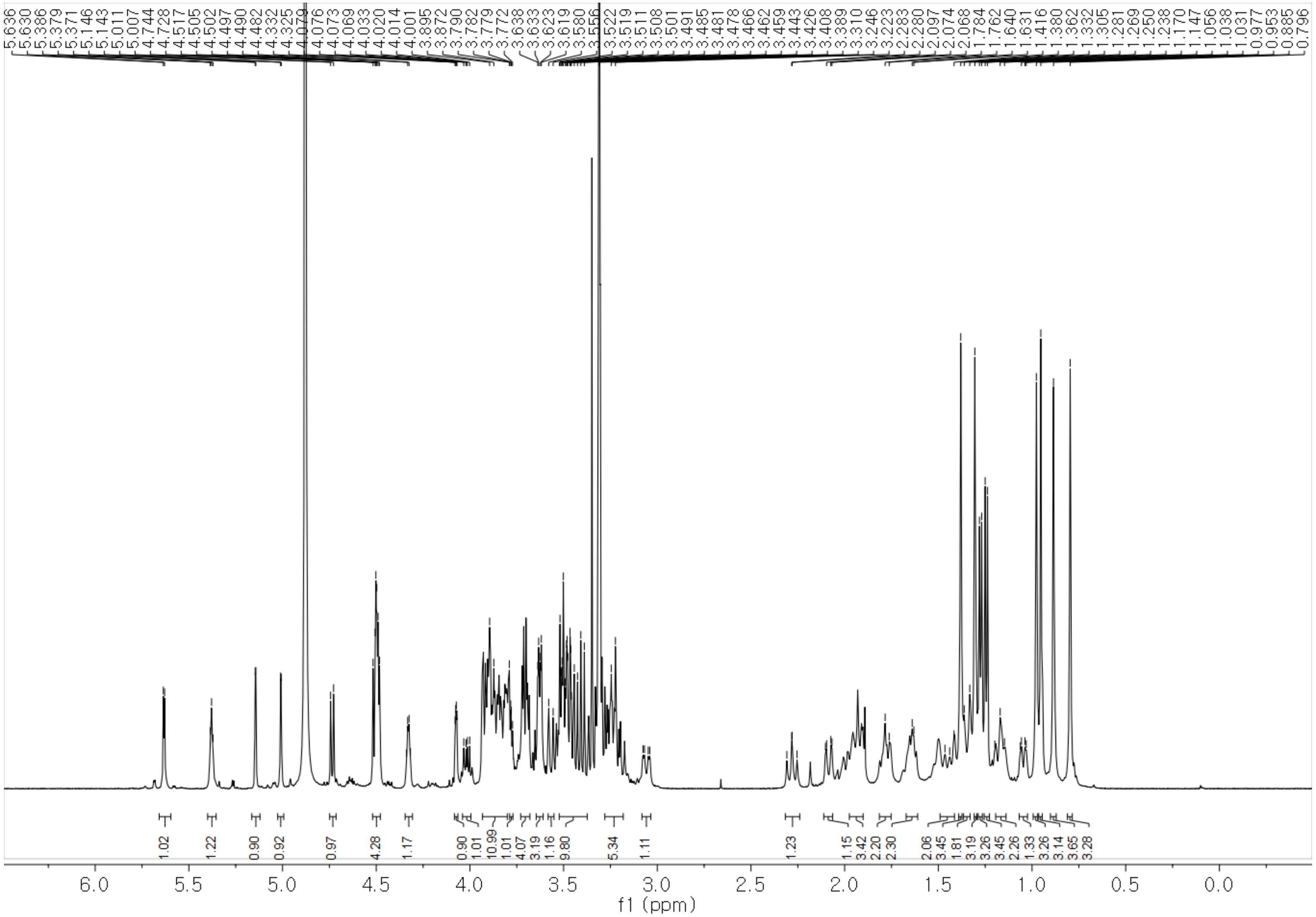

Figure S4. The ^{1}H NMR spectrum of compound **1** (500 MHz, CD_3OD).

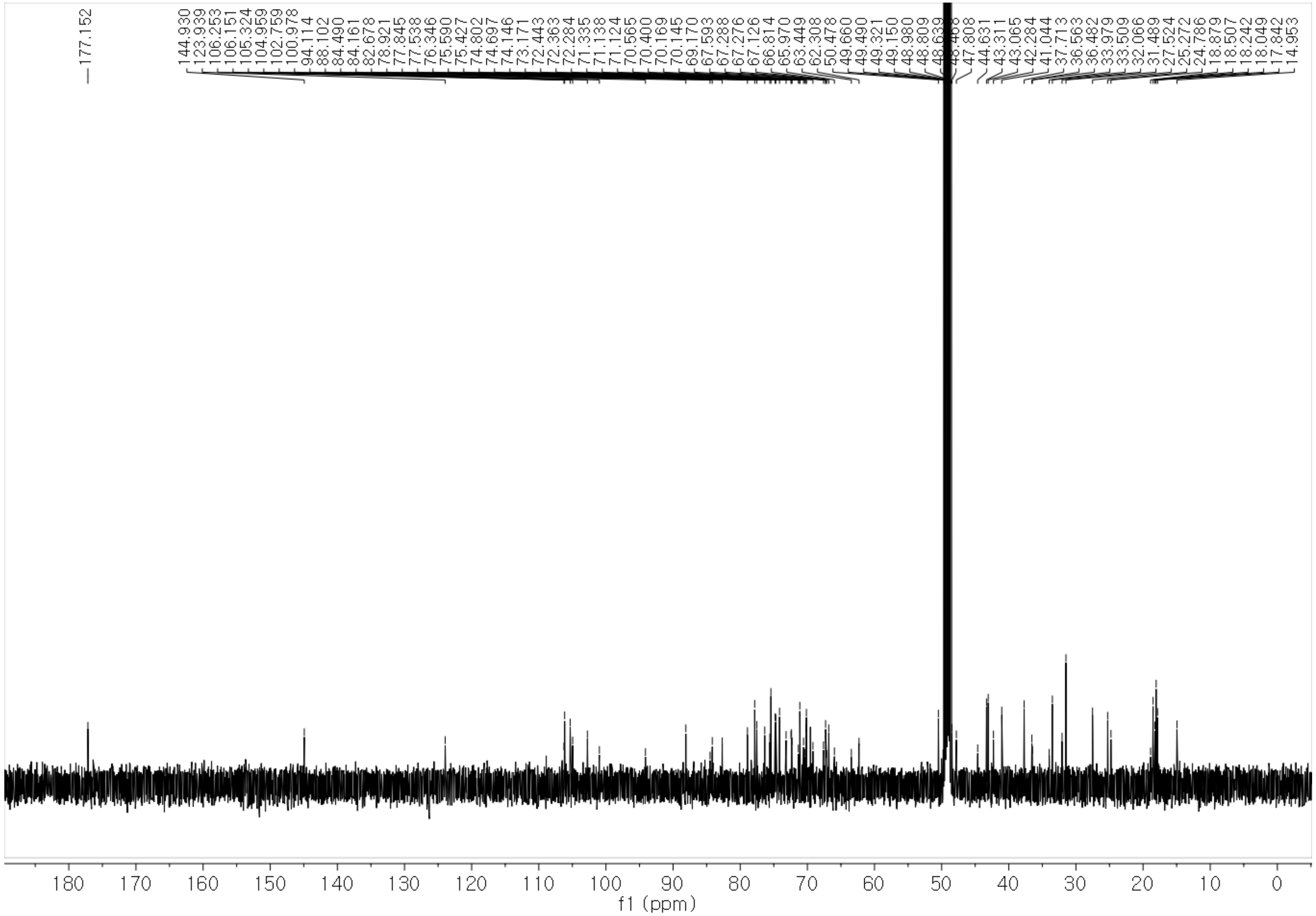

Figure S5. The ^{13}C NMR spectrum of compound **1** (125 MHz, CD_3OD).

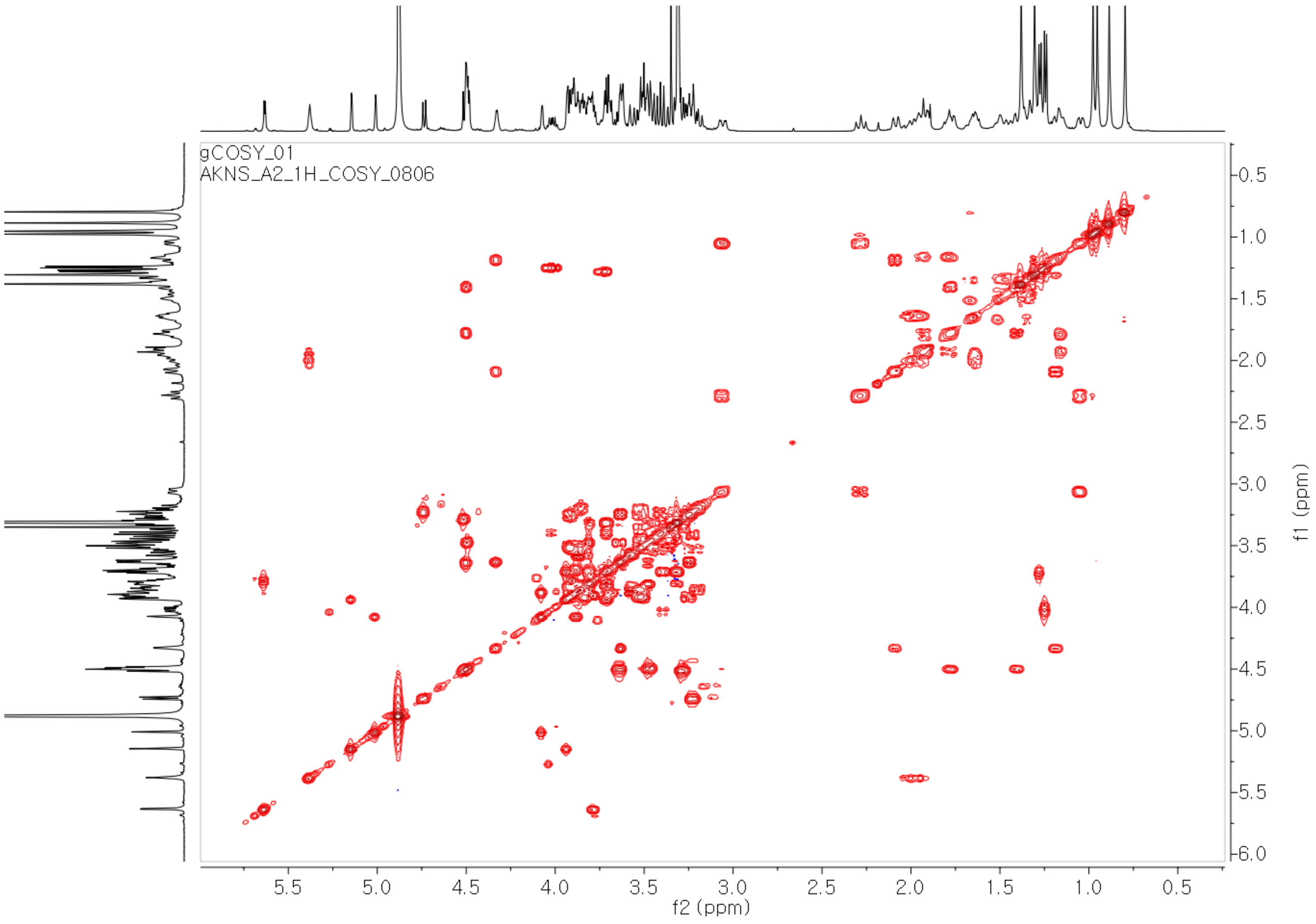

Figure S6. The ^{1}H-^{1}H COSY spectrum of compound **1** (500MHz, CD_3OD).

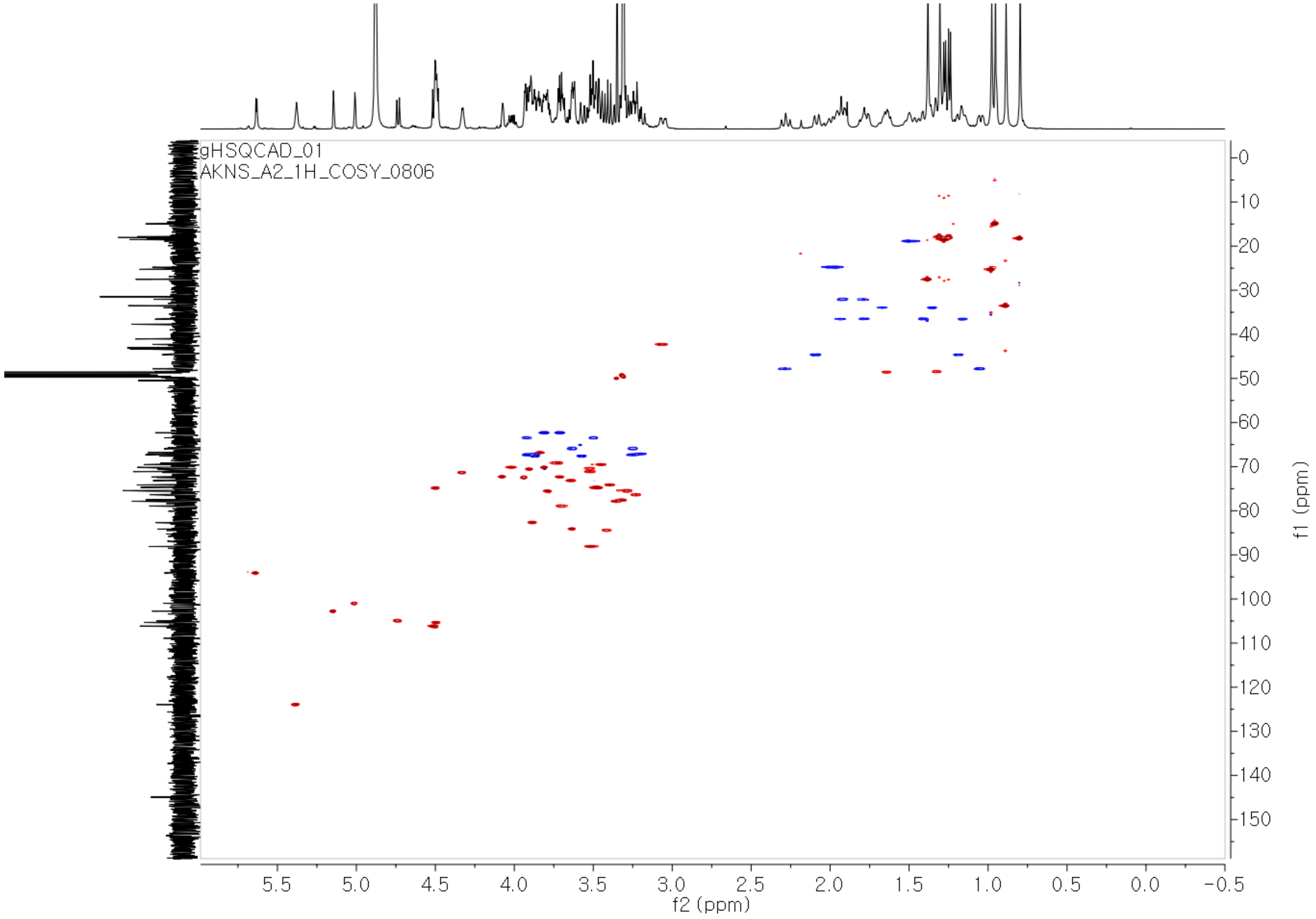

Figure S7. The HSQC spectrum of compound **1** (500 MHz, CD_3OD).

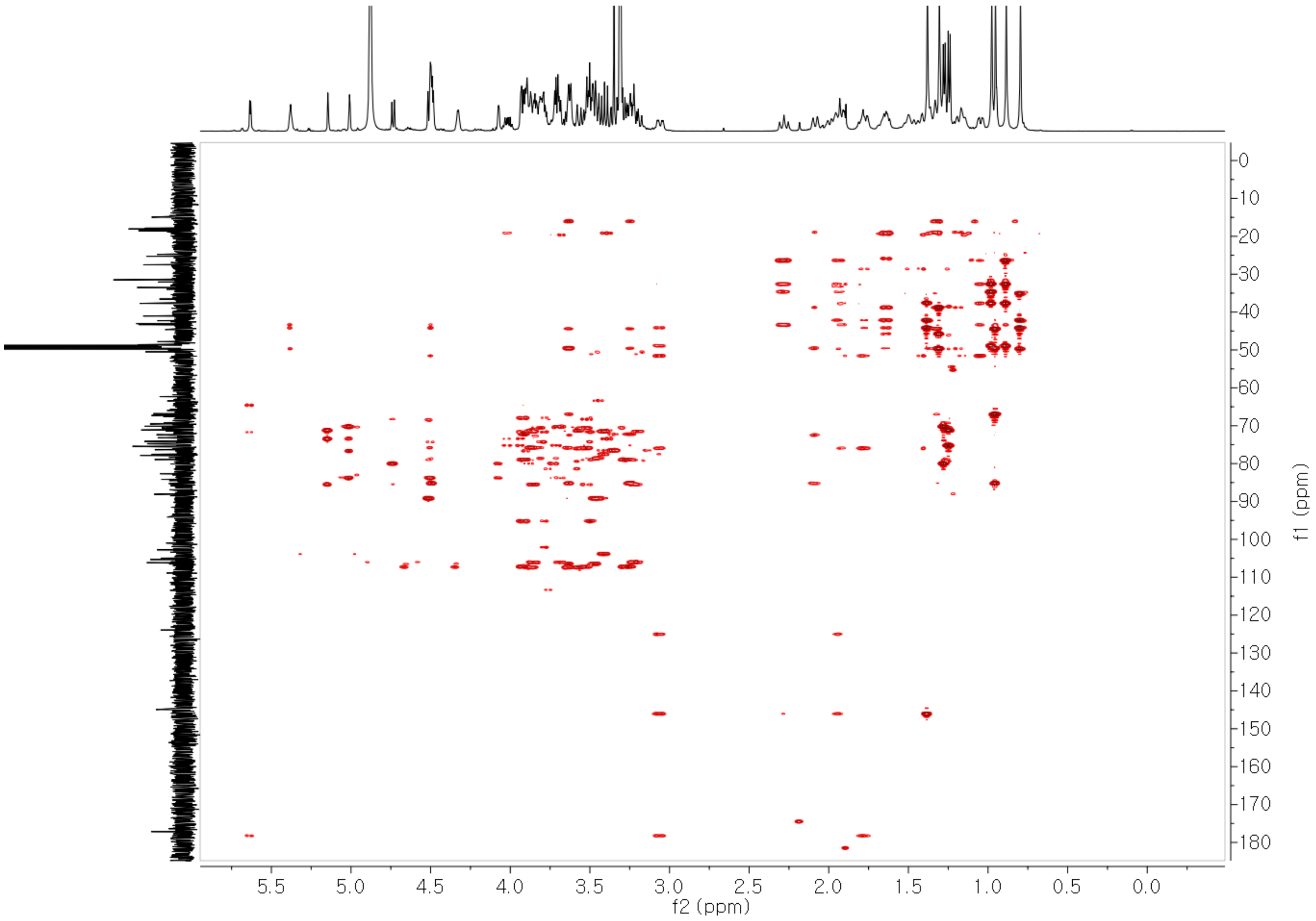

Figure S8. The HMBC spectrum of compound **1** (500 MHz, CD_3OD).

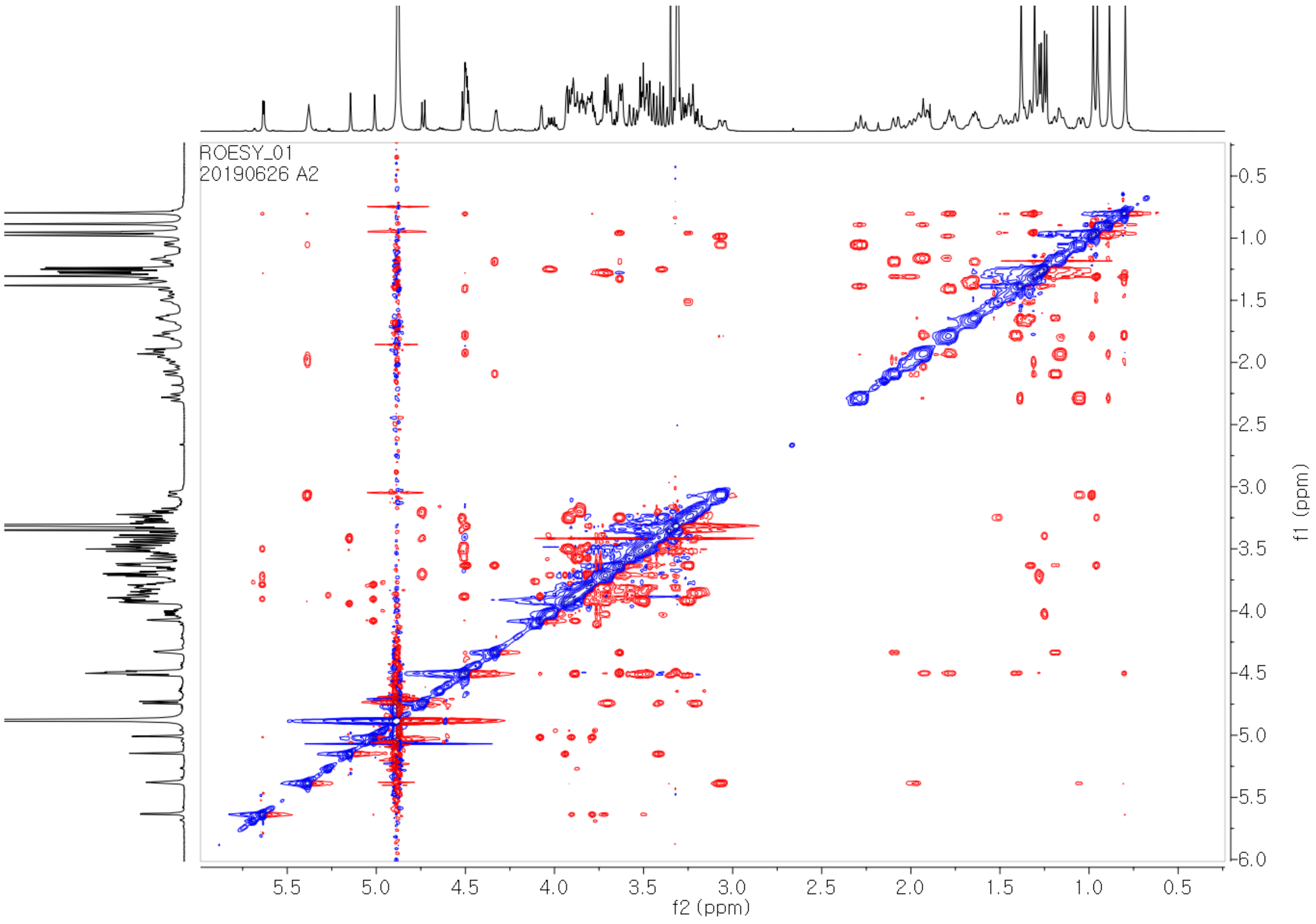

Figure S9. The 2D ROESY spectrum of compound **1** (500 MHz, CD_3OD).

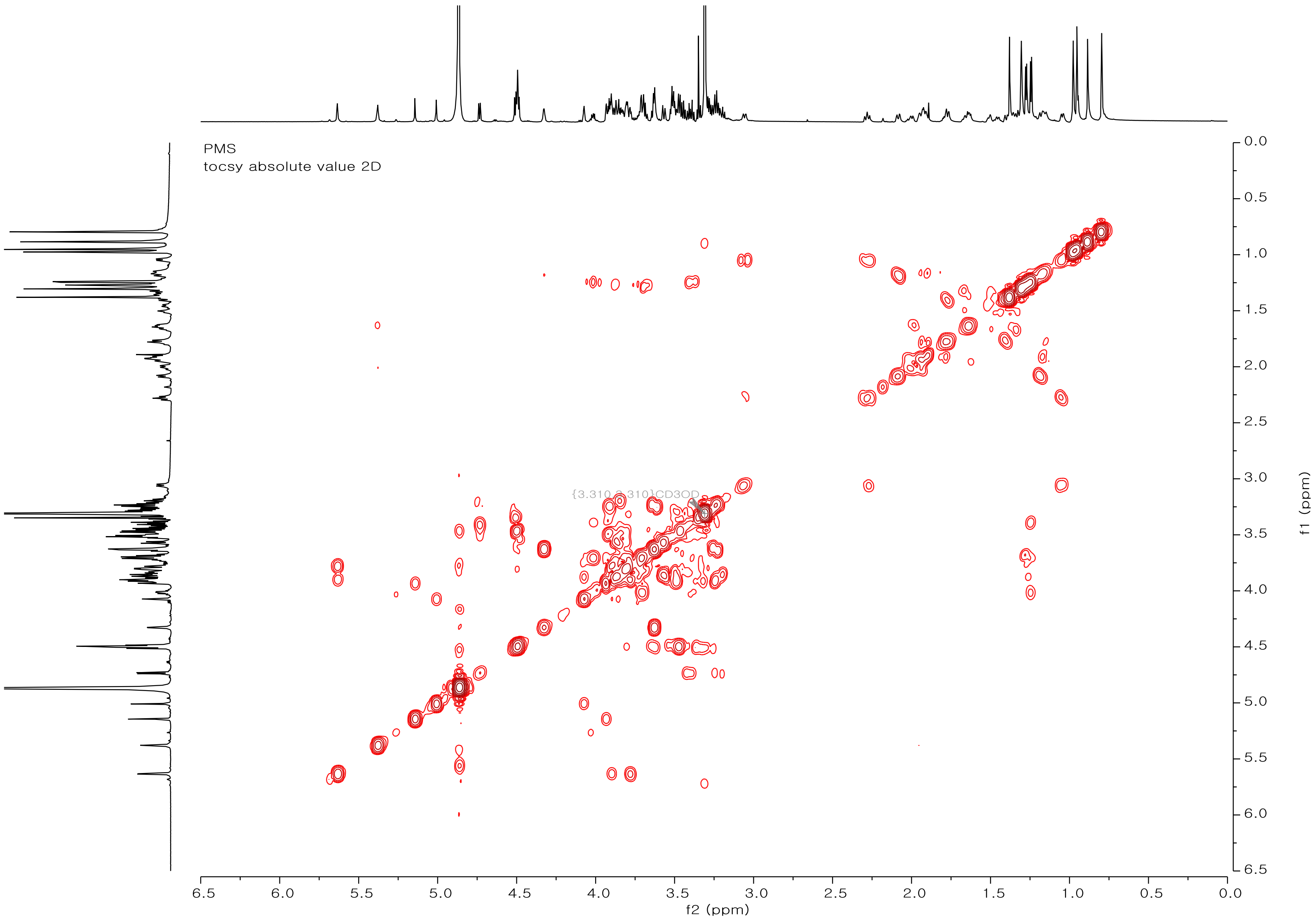

Figure S10. The 2D TOCSY spectrum of compound 1 (600 MHz, CD_3OD).

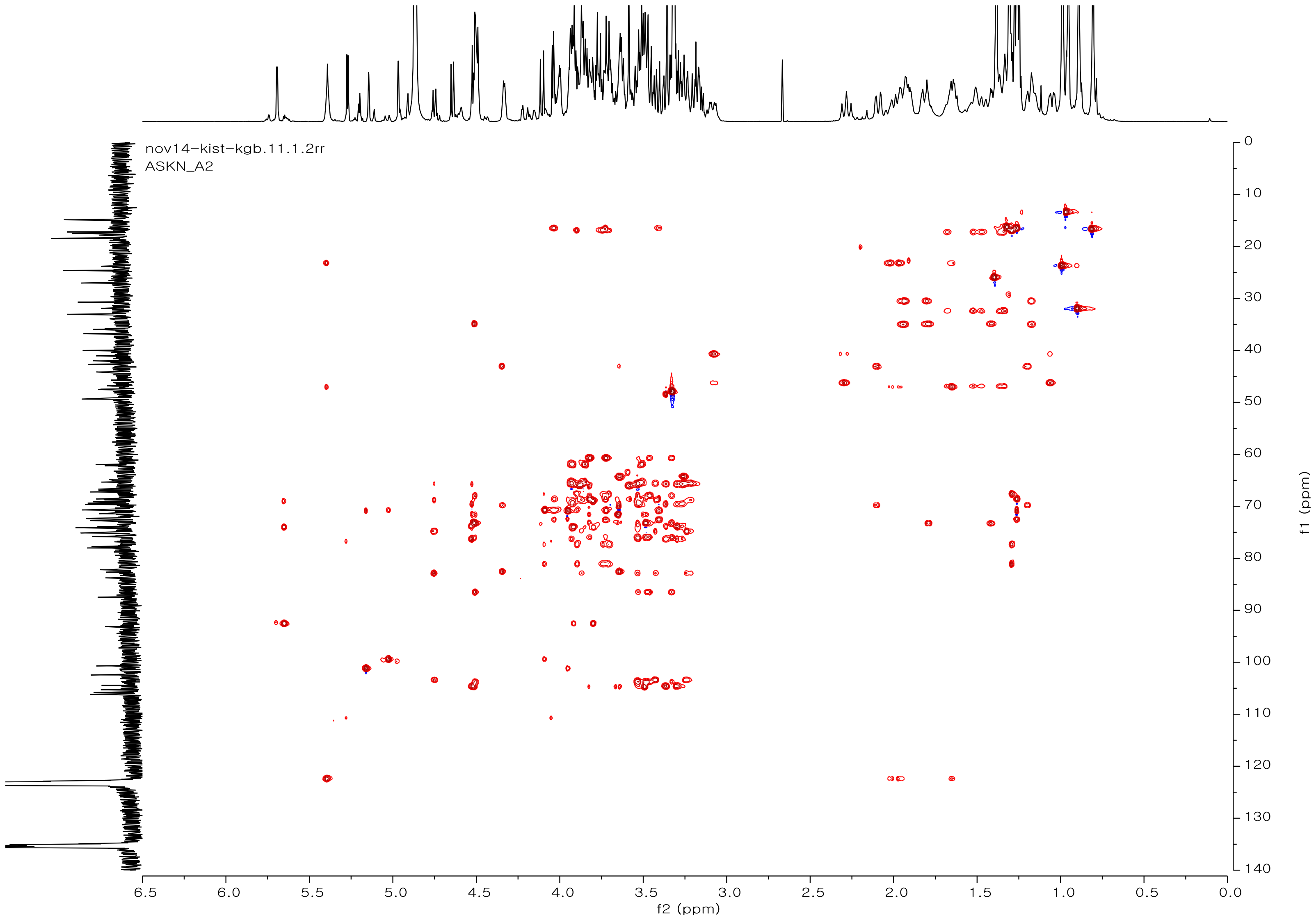

Figure S11. The TOCSY-HSQC spectrum of compound 1 (850 MHz, CD_3OD).

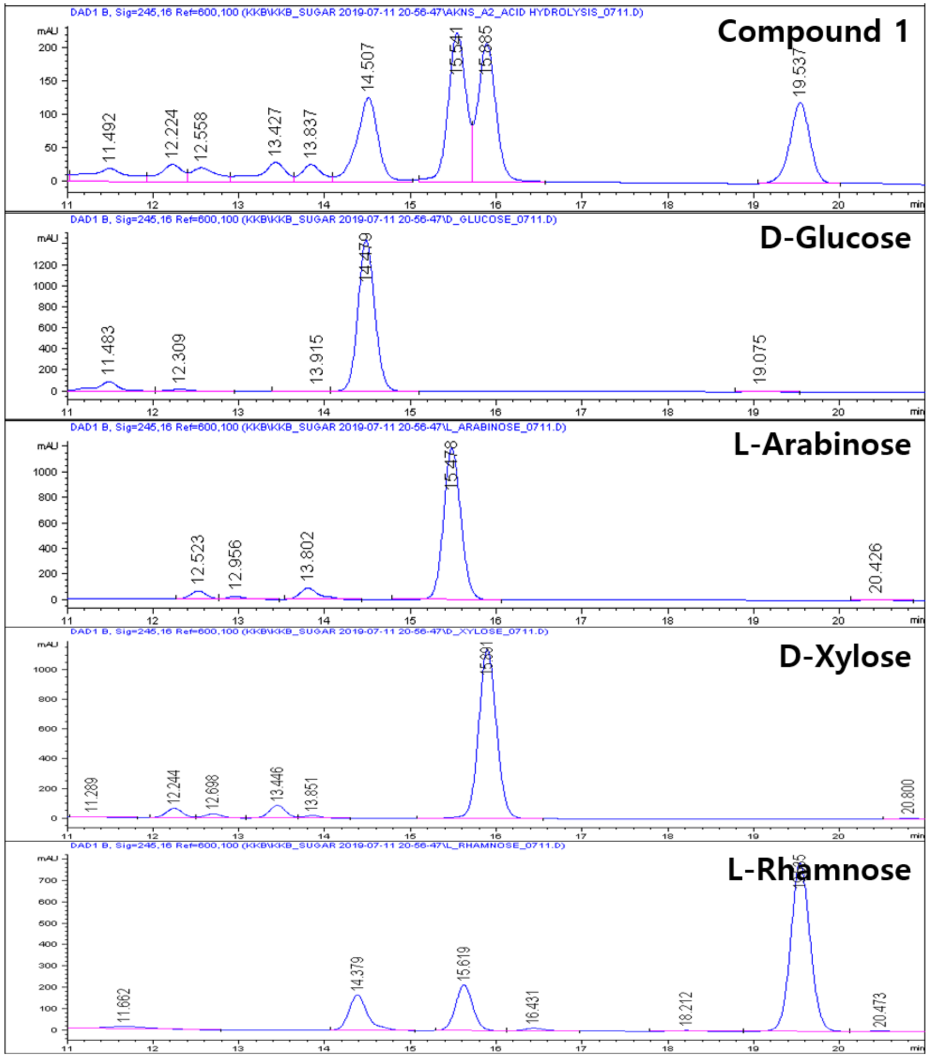

Figure S12. Sugar determination of compound **1**.

S13. ECD calculation method.

Conformational searches were performed by employing the procedure implemented in Spartan’14 software under the MMFF molecular mechanics force field, and the conformers were selected for geometry optimizations. Geometry optimizations were operated with DFT calculations at the B3LYP/6-31+G(d,p) level using Gaussian 09 package. TDDFT ECD calculations for the optimized conformers were performed at the CAM-B3LYP/SVP level with a CPCM solvent model in MeCN. The calculated ECD spectra were simulated with a half bandwidth of 0.3 eV, and the ECD curves were generated by SpecDis 1.64 software. The ECD spectra were weighted by Boltzmann distribution after UV correction.
